# Genome wide transcriptome analysis reveals ABA mediated response in *Arabidopsis* during gold (AuCl^−^_4_) treatment

**DOI:** 10.3389/fpls.2014.00652

**Published:** 2014-11-28

**Authors:** Devesh Shukla, Sneha Krishnamurthy, Shivendra V. Sahi

**Affiliations:** Department of Biology, Western Kentucky UniversityBowling Green, KY, USA

**Keywords:** Au, microarray, *Arabidopsis*, glutathione, plant hormone, secondary metabolism, transporters, ABA responsive element (ABRE)

## Abstract

The unique physico-chemical properties of gold nanoparticles (AuNPs) find manifold applications in diagnostics, medicine and catalysis. Chemical synthesis produces reactive AuNPs and generates hazardous by-products. Alternatively, plants can be utilized to produce AuNPs in an eco-friendly manner. To better control the biosynthesis of AuNPs, we need to first understand the detailed molecular response induced by AuCl^−^_4_ In this study, we carried out global transcriptome analysis in root tissue of *Arabidopsis* grown for 12- h in presence of gold solution (HAuCl_4_) using the novel unbiased Affymetrix exon array. Transcriptomics analysis revealed differential regulation of a total of 704 genes and 4900 exons. Of these, 492 and 212 genes were up- and downregulated, respectively. The validation of the expressed key genes, such as glutathione-S-transferases, auxin responsive genes, cytochrome P450 82C2, methyl transferases, transducin (G protein beta subunit), ERF transcription factor, ABC, and MATE transporters, was carried out through quantitative RT-PCR. These key genes demonstrated specific induction under AuCl_4^−^ treatment_ relative to other heavy metals, suggesting a unique plant-gold interaction. GO enrichment analysis reveals the upregulation of processes like oxidative stress, glutathione binding, metal binding, transport, and plant hormonal responses. Changes predicted in biochemical pathways indicated major modulation in glutathione mediated detoxification, flavones and derivatives, and plant hormone biosynthesis. Motif search analysis identified a highly significant enriched motif, ACGT, which is an abscisic acid responsive core element (ABRE), suggesting the possibility of ABA- mediated signaling. Identification of abscisic acid response element (ABRE) points to the operation of a predominant signaling mechanism in response to AuCl^−^_4_ exposure. Overall, this study presents a useful picture of plant-gold interaction with an identification of candidate genes involved in nanogold synthesis.

## Introduction

Gold, one of the least reactive metal in periodic table, possesses unique properties, namely, high free electron density, malleability and conductivity, and favors opportunities to produce stable and tuneable AuNPs for potential applications in diagnostics, biological imaging, sensors, therapeutic agent delivery, photodynamic therapy, catalysis, electronics etc. (Chen et al., 2008; Yeh et al., 2012; Spivak et al., 2013). Many chemical methods have been developed for the synthesis of AuNPs, but they produce hazardous toxic by-products, posing serious environmental health issues (Limbach et al., 2007). Alternatively, plant-based systems of nanomaterial synthesis were established recently to produce a range of AuNP shapes and sizes (Kumar and Yadav, 2009; Starnes et al., 2010; Geetha et al., 2013). Starnes et al. (2010) demonstrated *in planta* engineering of novel shapes in alfalfa by changing growth conditions of the plant. One of the current studies in *Arabidopsis* shows monodisperse spherical AuNPs formations in roots, grown hydroponically in the presence of 10 ppm of KAuCl_4_ (Jain et al., 2014).

Metallic gold with zero nutritive value does not cause toxicity to living organisms but higher concentrations of gold solutions (KAuCl_4_ or HAuCl_4_) may cause toxicity and impact plant growth negatively (Sharma et al., 2007; Starnes et al., 2010). Like other toxic heavy metals, for example, Cd, As, Hg, Pb, Au deactivates the proteins by binding to the sulfhydryl groups or disrupting the disulphide bonds and displacing the essential metal ion cofactors (Niemietz and Tyerman, 2002; Rodriguez et al., 2007).

A few investigations into the response of microorganisms to gold exposure have also been reported. For instance, Reith et al. (2009) characterized the mechanism of gold biomineralization in bacteria using a custom made microarray chip. Metal resistance genes, oxidative stress related genes and methyl transferases were reported to differentially express and cause reduction and detoxification of AuIII-complexes (Reith et al., 2009). Another interesting study demonstrated the involvement of a non-ribosomal small peptide—secreted as a secondary metabolite for the generation of solid gold particles from a gold resident bacterium *Delftia acidovorans* (Johnston et al., 2013).

Unlike the sufficient molecular information available with respect to heavy metals, little information is available in response to AuCl^−^_4_ exposure at whole transcriptome levels in plants (Taylor et al., 2014). Thus, the underlying mechanisms involved during the synthesis of AuNPs in plants remain largely elusive. To the best of our knowledge, the present study may be the first report of identifying the genes and mechanism operating in response to AuCl^−^_4_ treatment. This study will also help in predicting the changes in the associated metabolic networks.

In this study, 12 day old *Arabidopsis* root was exposed to chloroauric acid (HAuCl_4_) for 12 h, devoid of nutrient media, to record the specific response of Au. The principal finding of this study demonstrates that *Arabidopsis* senses the gold treatment as a strong stimulus, modulating expression of a total of 704 genes, which account for 2.5% of the whole transcriptome. The ABA- mediated signaling and glutathione binding appeared to be major possible mechanisms operating in response to AuCl^−^_4_.

## Materials and methods

### Plant material, growth conditions and treatments

Eighty seeds of the *Arabidopsis thaliana* cv. Col-0 were sterilized and germinated on mesh in each magenta box hydroponically in 0.5X MS medium for 5 days under a 16 h light (120 μmol m^−2^ s^−1^)/8 h dark photoperiod at 23°C (Jain et al., 2009). Thereafter, these seeds were transferred into 1X MS medium and grown for 7 days (Jain et al., 2009). The 12 day old seedlings were transferred to a 10 ppm AuCl^−^_4_ (HAuCl_4_, Sigma-Aldrich, USA) solution for 12 h at pH 4.2, in absence of nutrient medium. Another set of parallel experiments for control were carried out with distilled water at pH 4.2. Care was taken to ensure that only the roots came in contact with the 10 ppm gold solution. After 12 h of incubation in AuCl^−^_4_, the roots and shoots were separately collected for further experiment. Similarly, the *Arabidopsis* seedlings were treated with 10 ppm Ag^+^ [AgNO_3_; Acros, USA)] and 10 ppm Cu^2+^ [CuSO_4_.5H_2_O; Acros, USA] and the root and shoot tissues were collected for further experiments. Three independent biological replicates were used for each treatment and around 240 seedlings were used in each biological replicate to isolate RNA.

#### Total RNA isolation

Total RNA was isolated from frozen root and shoot tissues separately using RNeasy Plant Mini Kit with on column DNase digestion (Qiagen, USA), according to the manufacturer's instructions. The integrity of RNA was checked on agarose gel and concentrations were determined using Nanodrop (Wilmington, DE, USA).

### Transcriptome analysis

Affymetrix GeneChip® ara ST 1.0 GeneChips (.cel files submitted to GEO, accession number GSE55436) were used to carry out the microarray experiment. Target preparation, hybridization to arrays, washing, staining, and scanning were carried out following the instructions of the manufacturer (Affymetrix, USA). Three independent biological experiments were carried out for control (no treatment) and experimental (AuCl^−^_4_ treated) *Arabidopsis* seedlings. The hybridization data was processed using the Affymetrix GeneChip Command Console Software (AGCC version 3.2.4). Image files were analyzed to generate the probe intensity (.cel) files, using the default settings of AGCC. Microarray experiments were performed at the Microarray Core Facility of the University of Kentucky, in Lexington, Kentucky, USA. The probe set summarization (CHP) files for gene and exons were generated from feature intensity (.cel) files using RMA algorithms provided in the Expression Console v. 1.3. software. To identify statistically significant differentially expressed genes (DEGs) or exons, a combined criterion of greater than two-fold change was adopted with the ANOVA *p*-value (Condition unpair) <0.05 in the analysis. The normalized CHP files were analyzed using the secondary analysis tool Transcriptome Analysis Console, software to generate the.csv files containing DEGs.

#### Identification of transcription factor

The identification of transcription factors were carried out using a web based tool, a transcription factor database (http://arabidopsis.med.ohio-state.edu/AtTFDB/). A separate list of transcription factors with their sub-family name and synonyms was prepared and listed in **Table 2**.

#### GO enrichment analysis of DEGs

The singular enrichment analysis (SEA, http://bioinfo.cau.edu.cn/agriGO/index.php) was carried out on up- and downregulated genes. The TAIR AGI IDs with their affiliated GO annotations were subjected to agriGO analysis using the customized annotation mode. *Arabidopsis* TAIR 10 database was used as a background. The hieratical graphs and GO enrichment analysis table of DEGs were prepared, using a hypergeometric test with Bonferroni correction, at *P*-value 0.05, with 5 minimum number of mapping entries with complete GO gene ontology. The GO abundance chart was prepared manually by selecting the biologically relevant GO terms.

#### Quantitative RT PCR validation of transcriptome

The first strand cDNA was synthesized from 4 μg of total RNA in 20 μl reaction using Superscript III Reverse Transcriptase (Invitrogen Life Technologies, USA) using oligo(dT)_18_ primers, according to the manufacturer's instructions. The prepared cDNA was diluted five times and 1 μl (corresponding to 40 ng total RNA) was used to perform quantitative RT-PCR (qRT-PCR) reactions using the SYBR Green (Applied Biosystem) on 7300 Real –Time PCR system (Applied Biosystems). The reaction conditions for PCR and dissociation curves were as follows: 95°C for 10 min, 40 cycles of 95°C for 15 s, 58°C–60°C for 1 min and 95°C for 15 s, 60°C for 20 s, 95°C for 15 s, 60°C for 15 s, respectively. To normalize the gene expression, beta tubulin was used as an endogenous control. Wherever possible, most of the primers were designed manually from 3′ UTR of the gene and verified using Oligo Analyzer—1.1.2. The primer sequences are listed in Supplementary Table S1. The qRT-PCR experiment was repeated two times independently and in each experiment, three technical replicates were used. Relative gene expression was calculated using the 2^−ΔΔ CT^ values following Livak's method (Livak and Schmittgen, 2001).

#### Construction of heat map displaying expression profile of key genes under AuCl^−^_4_, As^V^, and Cd^II^

To differentiate the AuCl^−^_4_ specific response from toxic heavy metals, arsenate (As^V^) and cadmium (Cd^II^), 39 genes, selected on the basis of their high expression level (>5-fold) under Au exposure as well as their availability with Cd and As experiment, were analyzed using the Genevestigator perturbation tool (https://www.genevestigator.com/gv/plant.jsp). Log(2)-ratio change values of selected genes under As^V^ and Cd^II^ were obtained from Genevestigator program. A table was created by putting these values with the log(2)-ratio values of AuCl^−^_4_ and a heat map was constructed using an online graphing tool, plotly (https://plot.ly/plot). For the detail of the data please refer Supplementary File 4.

#### Analysis of biochemical pathways

The Plant MetGenMap, a web-based pathway analysis tool, was used to identify significantly changed biochemical pathways on the basis of expression profile data (http://bioinfo.bti.cornell.edu/cgi-bin/MetGenMAP/home.cgi). An input text file was prepared by putting the TAIR AGI IDs, linear fold change value and *P*-value as per the instruction given in Plant MetGenMap. The file was uploaded as an individual project and analyzed, as per the instructions given in the manual.

Similarly, another text file was created according to the format prescribed in Plant Metabolic Network (http://pmn.plantcyc.org/overviewsWeb/celOv.shtml) and uploaded at the Omics Data Viewer under the analysis, sub-portal of the Plant Metabolic Network.

#### Over-represented motif analysis in DEGs

The upstream sequences (TAIR 10 Loci Seq-1000 bp) belonging to up- and down-regulated genes were downloaded using the Bulk Data Retrieval tool in a Fasta format and saved as a tab delimited text file format (http://www.arabidopsis.org/tools/bulk/sequences/index.jsp). The search was carried out using the web based Motif finder tool: SCOPE (http://genie.dartmouth.edu/scope/). The parameters such as species, upstream sequence and strand search were selected as *Arabidopsis thaliana*, fixed 1000 bp and plus and minus strands both, respectively. Additionally, the TAIR Motif analysis tool (http://www.arabidopsis.org/tools/bulk/motiffinder/index.jsp) was also applied on the same data to further strengthen the results.

## Results and discussion

### Gold exposure significantly modulates transcriptome

**Figure 1:**
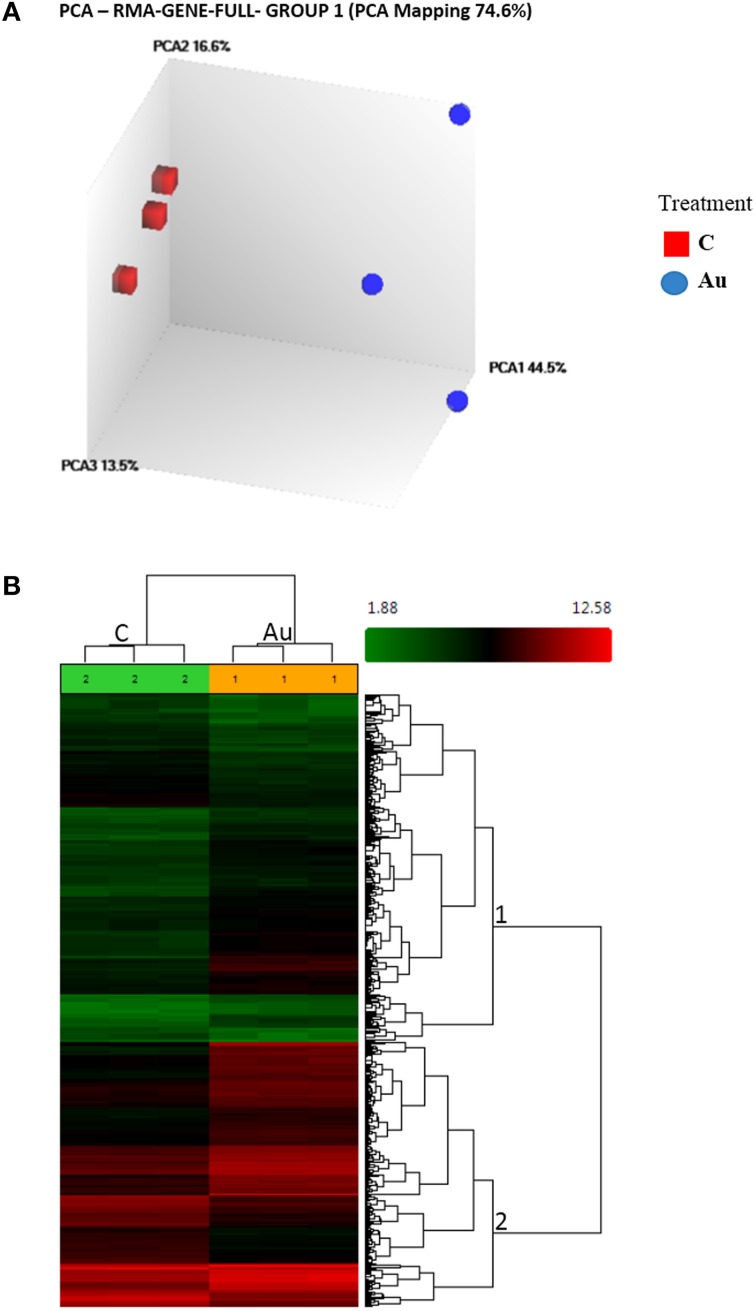
**Effect of gold (AuCl^−^_4_) treatment on the whole transcriptome in *Arabidopsis*. (A)** The Principal Component Analysis (PCA) shows remarkable differences between the control and treated samples. **(B)** The Hierarchical Clustering of the differentially expressed Transcript Clusters (TCs) under AuCl^−^_4_ (Condition1) vs. Control (Condition2). Number on the nodes displays major gene groups based on expression pattern. Color scale shows log_2_ signal intensity values.

In view of the rapid reduction of Au by sucrose or a nutrient ion like Fe^2+^, we believe, the use of a HAuCl_4_ solution devoid of nutrients would give us a precise insight into the interaction of plants with gold ions. Therefore, we isolated RNA from *Arabidopsis* plants exposed to 10 ppm gold solution at pH 4.2 (the natural pH of a gold solution) and microarray was performed using new unbiased Ara ST 1.0 chip on Affymetrix platform. After normalizing the image files using the RMA algorithm, principal component analysis revealed a distinct position of AuCl^−^_4_ treated samples from the control, suggesting a significant change in the transcriptome (Figure 1A). The microarray data was submitted to Gene Expression Omnibus (NCBI), with accession no. GSE55436. A One-Way ANOVA model was applied to the intensity data with adopted criterion: >2-fold change turning into a statistically significant (FDR ≤ 0.16) list of 704 genes (2.5%) and 4900 exons (2.8%) (Figure 1B, Table 1, Supplementary File 1). The high percentage of differentially regulated exons indicated occurrence of a number of alternative splicing events opening the opportunity to study it further (Supplementary File 2). The number of upregulated genes (nos. 492) observed were more than double of the number of downregulated genes (nos. 212), indicating the suitability of the exposed concentration (Table 1, Supplementary File 1).

**Table 1:** **Summary of transcriptome analysis**.

**S. No**.	**Query type**	**Genes**		**Exons**
1	Analysis type	Gene level differential expression analysis		Exon level differential expression analysis
2	Array Type		AraGene-1_0-st	
3	Genome version		tair10	
4	Annotation file	aragene-1_0-st-v1.na33.3.tair10.transcript.csv		aragene-1_0-st-v1.na33.3.tair10.transcript.csv
				aragene1_0-st-v1.na33.2.tair10.probeset.csv
5	Total number	28403		171941
6	Differentially expressed	704		4900
7	Up regulated (Exp. Vs. Con)	492		2690
8	Down regulated (Exp. Vs. Con)	212		2210
9	Algorithm		One-Way between-subject ANOVA (unpaired)	
10	Default filter criteria 1		Fold change (linear) < -2 or fold change (linear) > 2	
11	Default filter criteria 2		ANOVA *p*-value (condition pair) < 0.05	

### General annotation of DEGs in response to AuCl^−^_4_

We described the functional roles of genes that shared a similar family and/or category on the basis of available literature and annotations. Glutathione-S-transferases (GSTs) were the major detoxifying enzymes that catalyze the conjugation of reduced glutathione to different cytotoxic substrates and subsequently transport into the vacuole in an ATP-dependent manner. These genes are induced by a number of metabolic processes, as well as in response to stress, heavy metals or plant hormones (auxin/jasmonic acid) (Mars, 1996; Chakrabarty et al., 2009). Additionally, some GSTs also showed glutathione peroxidase activity, strengthening their role in antioxidant metabolism (Mars, 1996). Nineteen members belonging to the GSTs family were differentially regulated (Supplementary File 1). Most of the members belong to GST-tau and GST-phi, GST-lambda class (Wagner et al., 2002). These proteins were classified under multiple GO categories, for example, metabolic process, cellular process, response to stimulus or stress or biotic stimulus, secondary metabolic process, catabolic process and lipid metabolic process (Supplementary File 3). Members of Phi class, were reported to show Co or Cu ion binding activity (Supplementary File 3). An important member of GST phi class, AT1G49860 (GSTF14), was found to be downregulated by 4.4-fold, and involved in anion transport, lateral root development, and root hair cell differentiation (Supplementary File 1). Altogether, these observations suggest a possibility of GSH-based detoxification and reduction of ionic Au species into 0 valent non-ionic, non-reactive form.

Seven members of beta-glucosidase gene family were upregulated at varying levels (Supplementary File 1). The expression patterns of these classes of genes were reported to alter according to the change in the level of sugar inside the cell (Lee et al., 2007). The beta-glucosidase 30 was expressed at the highest level (26-fold) and known for hydrolysing o-glycosyl compounds and modified cell walls (Fujiki et al., 2001). Additionally, deglucosylating activities of these enzymes may regulate the activity of a glucosylated forms of a plant hormone. Three other sugar transporters, members of the ERD6 family, were downregulated (Supplementary File 1). These observations suggest that the gold (AuCl^−^_4_) exposure could change the level of sugar inside the cell, triggering sugar associated signaling.

Cytochrome P450 is a stress responsive super gene family, comprising 267 members in *Arabidopsis*, and plays a variety of roles in biological processes like oxidation-reduction and detoxification of xenobiotics (Chakrabarty et al., 2009; Liu et al., 2010). These proteins share a class under metabolic, cellular process and response to stress or stimulus. Twenty members of this family were differentially regulated, in which 13 were up- and 7 were downregulated (Supplementary File 1). The highest expression was 22.7-fold for cytochrome P450, family 82, subfamily C, polypeptide2 (AT4G31970), which was known to induce under hypoxia condition and shows jasmonate-induced root growth inhibition, electron carrier, oxido-reductase and oxygen binding activity (Liu et al., 2010).

Four auxin response related genes were upregulated, signifying AuCl^−^_4_-induced auxin signaling (Supplementary File 1). AT1G60750 locus encodes an auxin-induced atb2-like protein involved in metabolic processes such as oxidation and reduction. AT5G13370, an auxin responsive GH3 family protein, was reported to induce in response to auxin stimulus (Takase et al., 2003). AT1G76520, an auxin efflux carrier protein, possesses auxin-hydrogen symporter activity and is involved in auxin polar transport and localization (Barbez et al., 2012). Recently, in one of our studies we showed the progressive reduction of primary root length and meristematic activity of primary and lateral roots under higher concentrations of AuCl^−^_4_ (Jain et al., 2014). Overall, the upregulation of these genes suggest the importance of AuCl^−^_4_ mediated-auxin response, which has the capacity to modulate the root system architecture (Jain et al., 2014).

Fourteen different methyl transferases (MTs) were differentially regulated. Six members of S-adenosyl methionine (SAM) -dependent MTs, two members of O-methyl-transferase protein and two others were upregulated (Supplementary File 1). SAM is a well-known methyl donor, suggesting the significance of the methylation event in response to gold. These results are in agreement with an earlier study in which a microorganism exposed to AuCl^−^_4_ exhibited upregulation of several MTs, suggesting the possibility of Au methylation as a conserved detoxification mechanism (Reith et al., 2009).

Three members of ABC transporter G family were upregulated (Supplementary File 1). One member, PDR12, was reported to be induced in response to auxin, ABA, ethylene, cyclopentenone, jasmonic, and salicylic acid (Lee et al., 2005; Kang et al., 2010). Additionally, it was known to take part in transportation of Pb, ABA, and terpenoids, revealing some new possibilities with respect to AuCl^−^_4_ transport (Lee et al., 2005; Kang et al., 2010).

A large gene family of at least 56 members encoding MATE-related proteins was identified from the *Arabidopsis* genome (Tiwari et al., 2014). MATE are known to play various roles, such as disease resistance, trafficking of secondary metabolites into the vacuole, and exporting out the small organic molecules like citric acid to sequester metal ions or iron transport and distribution in the plants (Tiwari et al., 2014). In the present study, five members of the MATE gene family were upregulated (Supplementary File 1). Of these, an important gene, ATDTX1, was earlier identified as a detoxifying efflux carrier exporting toxic compounds outside the cell (Li et al., 2002).

Sulfate transporters are shown to be induced under sulfur starvation, as well as against the exposure of heavy metals (Chakrabarty et al., 2009). Sulfur is the vital part of S-rich non-protein thiols like GSH and phytochelatins, known to exhibit heavy metal tolerance and accumulation (Shukla et al., 2012, 2013a,b). Interestingly, the expression of two members of the phytochelatin synthase (PCS) gene family was modulated <2-fold in the present study, indicating the role of PCs in the detoxification of Au (data not shown). In this case, sulfate transporter 3.1 encoding a chloroplast localized transmembrane protein was also upregulated by 6.5-fold (Supplementary File 1), similar to that reported by Cao et al. (2013). Another member, sulfate transporter 3.5 was upregulated by 4.8-fold and known to be involved in root to shoot transport of sulfates. Induction of these transporters may suggest the enhanced requirement of S-containing non-protein thiols against the oxidative stress caused by AuCl^−^_4_ exposure.

A phosphate transporter 3.2, which encodes a mitochondrial phosphate transporter 2 (MPT2), reported to be induced in response to ethylene stimulus, was upregulated (Supplementary File 1). Nodulin, a group of MTN21-like transporter family proteins involved in the transport of multiple amino acids, were upregulated (Supplementary File 1). Similarly, a transmembrane amino acid transporter, (AT1G08230), which codes for an H^+^ driven, high affinity gamma-aminobutyric acid (GABA) transporter localized at plasma membrane, was upregulated (Supplementary File 1). Some putative oligo-peptide transporters (OPTs), known for the transport of peptides/nitrates having the probability of metal binding, were upregulated (Supplementary File 1). Interestingly, two heavy metal associated domain containing transporters, were also up- and downregulated. It appears that the treatment of gold (Au) also upregulated some transporters, which are relatively new or less characterized.

### Identification of transcription factors from DEGs

In order to shed light on the regulatory genes modulated by the AuCl^−^_4_ treatment, we separated the list of transcription factors from DEGs using an *Arabidopsis* transcription factor identification web-based tool (http://arabidopsis.med.ohio-state.edu/AtTFDB/). We found a total of 57 transcription factors that were differentially expressed in the present experiment (Table 2). The high number of expressed regulatory proteins in this experiment indicates their important role in transcriptome modulation. Of these, the maximum number (10) was represented by the members of AP2 domain containing ethylene-responsive element binding proteins (AP2-EREBP) and NAC domain containing proteins. Generally, the AP2EREBP gene family was known to be involved in plant responses to drought, cold, salt, and ABA signaling (Mishra et al., 2013, 2014). Interestingly, a member of the AP2-EREBP family (At5g64750), was reported to induce by ABA treatment but functions as a transcriptional repressor of ABA response (Pandey et al., 2005). Members of the NAC gene family were reported to associate with plant senescence (Shibuya et al., 2014). Recently, Christiansen and Gregersen (2014) showed that the NAC TF binds with the CGT motif. It is worth stating that CGT motif shares a part with the abscisic acid responsive core element (ABRE), ACGT. Six members of the R2R3-MYBs gene family were differentially expressed in *Arabidopsis*; these are known to be involved in abiotic stress response. Five members of the WRKY gene family were differentially regulated as well, of which WRKY70 and WRKY63 were earlier reported to be involved negatively in leaf senescence and ABA response, respectively (Ren et al., 2010; Besseau et al., 2012). WRKY8, and WRKY31 were reported to be involved in jasmonate signaling (Schluttenhofer et al., 2014). WRKY28 is responsive to drought, salt, and oxidative stress (Tripathi et al., 2014). Some other members of different regulatory gene families were also expressed, such as C2H2 zinc-finger, Homeobox HDZIP, bHLH, C3H, Trihelix, C2C2-CO-like and GRAS etc. Overall, a common signaling event associated with these regulatory proteins is senescence. Hence, it may be possible that abscisic acid (ABA) is playing a key role in mediating plant responses against the AuCl^−^_4_ challenge.

**Table 2:** **List of transcription factors regulated during AuCl^−^_4_ treatment**.

**TF family name**	**TF locus Id**	**Protein name**	**Sub family**	**Gene name, synonym**
AP2-EREBP	At1g71520		NA	
AP2-EREBP	At5g64750		NA	ABR1
AP2-EREBP	At2g38340		NA	
AP2-EREBP	At2g33710		NA	
AP2-EREBP	At2g40340		NA	
AP2-EREBP	At1g22810		NA	
AP2-EREBP	At1g22985		NA	
AP2-EREBP	At3g23230		NA	
AP2-EREBP	At3g11020		NA	DREB2, DREB2B
AP2-EREBP	At4g13620		NA	
NAC	At1g02230		NA	ANAC004
NAC	At1g52890		NA	ANAC019
NAC	At5g39610		NA	ANAC092, ATNAC2, ATNAC6
NAC	At2g43000		NA	anac042
NAC	At3g15500		NA	ANAC055, ATNAC3
NAC	At1g77450		NA	anac032
NAC	At5g63790		NA	ANAC102
NAC	At1g28470		NA	ANAC010, SND3
NAC	At4g27410		NA	ANAC072, RD26
NAC	At5g39820		NA	anac094
MYB	At1g68320	AtMYB62	R2R3-MYBs	AtMYB62, BW62B, BW62C, MYB62
MYB	At1g48000	AtMYB112	R2R3-MYBs	AtMYB112, MYB112
MYB	At2g47190	AtMYB2	R2R3-MYBs	ATMYB2, MYB2
MYB	At5g14750	AtMYB66	R2R3-MYBs	ATMYB66, WER, WER1
MYB	At4g01680	AtMYB55	R2R3-MYBs	AtMYB55, MYB55
MYB	At4g25560	AtMYB18	R2R3-MYBs	AtMYB18, LAF1
WRKY	At4g22070	AtWRKY31	NA	AtWRKY31, WRKY31
WRKY	At1g66600	AtWRKY63	NA	AtWRKY63, WRKY63
WRKY	At4g18170	AtWRKY28	NA	AtWRKY28, WRKY28
WRKY	At5g46350	AtWRKY8	NA	AtWRKY8, WRKY8
WRKY	At3g56400	AtWRKY70	NA	ATWRKY70, WRKY70
bHLH	At4g25400	AtbHLH118	NA	
bHLH	At1g62975	AtbHLH125	NA	
bHLH	At4g20970	AtbHLH162	NA	
C2C2-CO-like	At4g27310		NA	
C2C2-CO-like	At5g57660		NA	COL5
C2C2-CO-like	At5g54470		NA	
C2C2-Dof	At1g69570		NA	
C2H2	At5g60470		NA	
C2H2	At3g28210		NA	PMZ
C2H2	At2g21400		NA	SRS3
C2H2	At3g49930		NA	
C2H2	At3g05155		NA	
C2H2	At3g05160		NA	
C3H	At5g05530		NA	
C3H	At5g42200		NA	
C3H	At1g14200		NA	
C3H	At5g55970		NA	
Homeobox	At1g70920		HD-Zip II	ATHB18
Homeobox	At3g61890	AtHB12	HD-Zip I	ATHB-12, ATHB12
Trihelix	At5g01380		NA	GT-3a
Trihelix	At1g12040		NA	LRX1
MADS	At1g17310		TypeI	AGL100
NLP	At2g43500	AtNLP8	NA	
RAV	At3g25730		NA	
G2-like	At5g45580		NA	
GRAS	At3g13840	AtGRAS16	NA	

### Singular enrichment analysis (SEA) of DEGs

**Figure 2:**
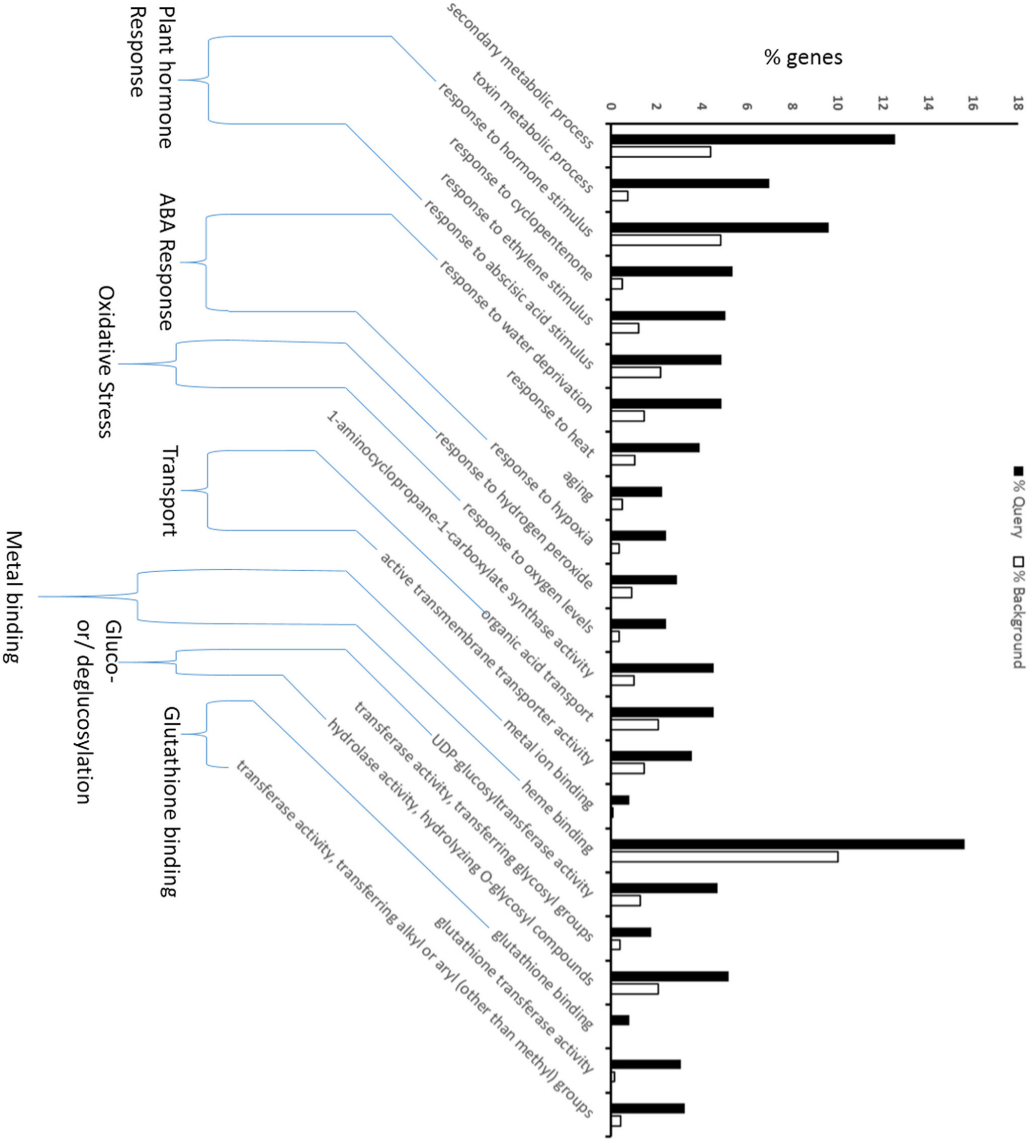
**GO enrichment analysis for differentially regulated genes during gold treatment (AuCl^−^_4_)**. The analysis shows the ABA and Glutathione related processes were affected. The Singular Enrichment Analysis (SEA) of up- and downregulated genes with their GO IDs was performed using the agriGO analysis tool under customized mode, *P* < 0.05 and minimum 5 mapping entries. The black bar shows the fraction of query genes for particular GO term out of total no. of query genes, and the white bar provides the similar information with respect to genes present in background (whole genome). Please see details in Materials and Methods.

To discern the relevant biological meaning, SEA was performed on the DEGs using an AgriGO analysis tool (Supplementary File 3). Seventy-nine significantly (FDR < 0.04) enriched GO terms were grouped under three functional categories: biological process (BP); cellular component (CC); and molecular function (MF) (Supplementary Figure 1). Of these, 23 GO terms, which were biologically relevant with the present experiment and objective, were displayed in Figure 2. The response of plant hormones, such as cyclopentenone, an intermediate of jasmonic acid, ethylene, and abscisic acid, appears to be upregulated. The ABA associated response, oxidative stress, metal ion binding, transport and glutathione binding were also found to be upregulated. This data suggest that AuCl^−^_4_ treatment may trigger ABA specific response, which could potentially modulate the expression of large set of genes. Water deprivation, heat, hypoxia and aging are associated with the ABA-mediated signaling, strengthening the ABA specific response. Moreover, ethylene and ABA do cross talk with each other at several points and together may generate these responses. Toxicity of gold solution may produce oxidative stress similar to other toxic heavy metals, and upregulation of H_2_O_2_ response is a sign of oxidative stress. The plant produces non-enzymatic antioxidant molecules such as GSH, ascorbic acid and secondary metabolites to combat the oxidative stress. In this study, glutathione appeared to be a key player for reducing the oxidative stress. A significant upregulation of GST activity opens the possibility of conjugation of GSH with toxic metabolites like ROS, or possibly AuCl^−^_4_, to sequester them into the vacuole for detoxification (Figure 2).

### Comparative expression profile of key genes under AuCl^−^_4_ and toxic heavy metal

**Figure 3:**
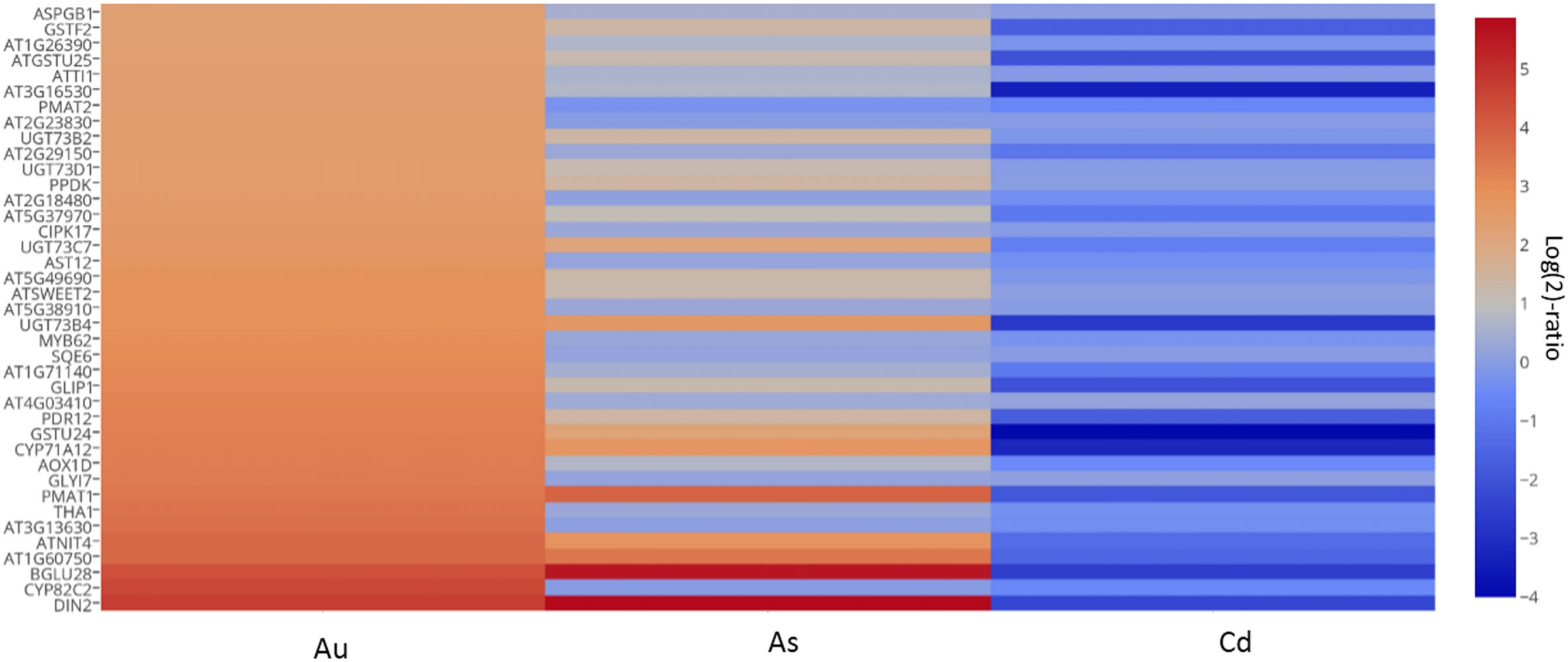
**Expression profile of higly expressed genes demonstrates a distinct Au response in comparison to As^V^ and Cd^II^**. Thirty nine genes, selected on the basis of their expression cut off (>5-fold) under the conditions of Au and heavy metal exposures, demonstrate significant differences in expression patterns under the three conditions. The log(2)-ratio expression values for As^V^ and Cd^II^ downloaded from the known database using the Genevestigator were combined with the values from the present data and a heat map was prepared using an online plot tool. Please refer to the Materials and Methods for more information.

The effect of AuCl^−^_4_ on plant gene expression is largely elusive, however, the research on plant-based synthesis of Au nanoparticles has started to show results. In order to differentiate the AuCl^−^_4_ specific genetic response from toxic heavy metal(loid)s like arsenic (As) and cadmium (Cd), we constructed a heat map by taking 39 genes on the basis of high expression cut off (>5-fold) under AuCl^−^_4_ exposure as well as their presence under Cd and As exposure (Supplementary File 4). In general, all these genes were highly upregulated in the presence of AuCl^−^_4_. Contrastingly, they were found to be downregulated in presence of As^V^ or Cd^II^ (Figure 3). These genes showed a significantly distinct expression profile in the presence of AuCl^−^_4_, compared to As^V^ and Cd^II^, suggesting that the interaction of AuCl^−^_4_ with plants is different. Since Au ions or nanoparticles possess distinct—physicochemical properties, it may generate a unique signature on the plant transcriptome.

### Validation of array data using qRT-PCR under AuCl^−^_4_, as well as for Ag and Cu exposure

**Figure 4:**
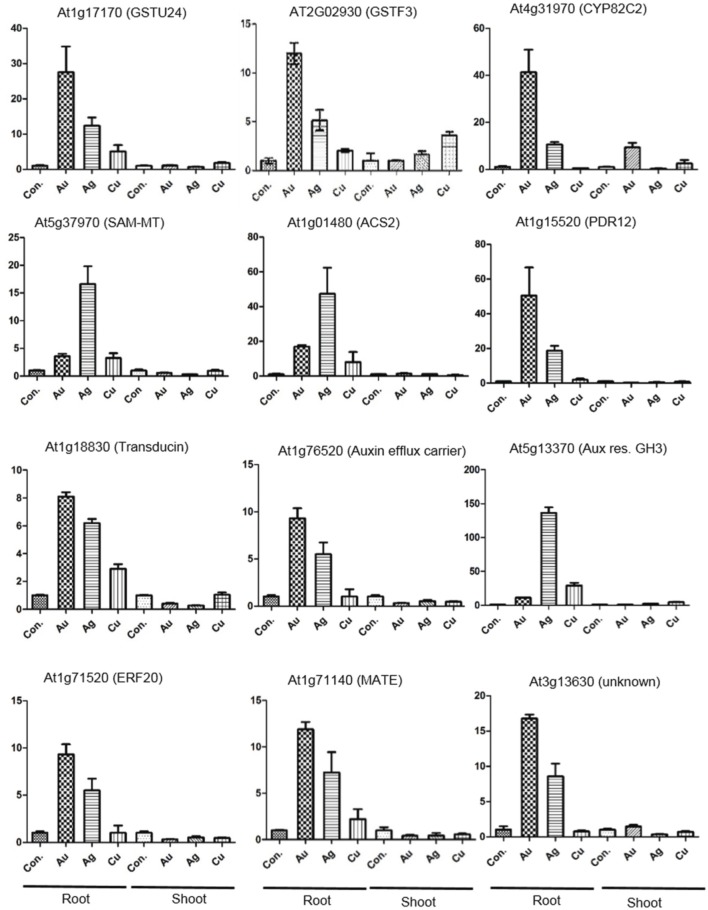
**Validation of the array data through qRT-PCR**. The expression analysis of the selected genes shows the positive correlation with the microarray data. qRT-PCR was carried out with root and shoot tissues of *Arabidopsis* exposed to AuCl^−^_4_, Ag^+^, and Cu^2+^, under similar experimental conditions used for the microarray analysis.

Twelve key genes, were selected on the basis of their possible role in Au- reduction, detoxification, plant hormone responses, signaling, transport, transcriptional regulation and unknown function for the validation of microarray data through quantitative RT-PCR (Figure 4). We performed the qRT-PCR separately for the root and shoot tissues to resolve tissue-specificity. Including Au, we chose metals sharing similar group in the periodic table (i.e., Ag, Cu) to test the metal specificity. The quantitative gene expression results correlated with the trend of regulation observed in the microarray experiment. In general, the expression level of genes was higher in root as compared to shoot (Figure 4). The transporters (PDR-12, MATE), and GSTs were more responsive to AuCl^−^_4_, compared to Ag and Cu, indicating probably their role in Au reduction or detoxification (Figure 4). Cytochrome P450 (AT4g31970, CYP82C2) was upregulated significantly in response to AuCl^−^_4_, indicating their involvement in detoxification (Figure 4). This enzyme possesses properties like oxidoreductase activity, acting on paired donors, with incorporation or reduction of molecular oxygen (Supplementary File 3). Expression of an auxin efflux carrier (AT1G76520) suggests the triggering of auxin mediated signaling. Expression of ethylene biosynthesis and signaling related genes: ACS2 (AT1G01480) and ERF20 (AT1G71520) points toward the Au-mediated induction of ethylene and ABA signal transduction (Figure 4). Surprisingly, a beta subunit of G protein, named here as transducin (AT1G18830), was upregulated in response to AuCl^−^_4_, Ag and Cu in a decreasing order (Figure 4). G protein coupled receptors are known to be involved in ABA reception (Nitta et al., in press). It can be hypothesized that the plant uses an ABA receptor, associated with G protein, and transduces signaling, modulating the expression of a large set of genes for adaptation purpose under AuCl^−^_4_ exposure (Yadav et al., 2013, 2014).

### Prediction of plant metabolic shift in response to AuCl^−^_4_

**Figure 5:**
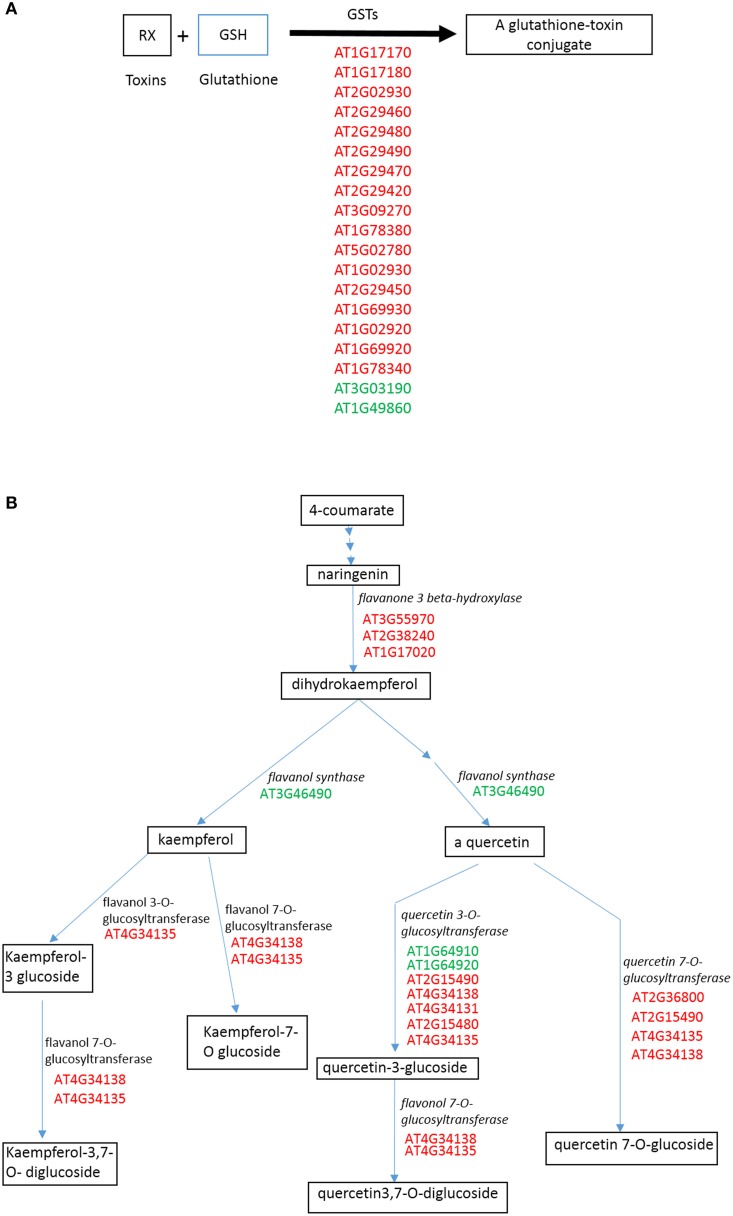
**Prediction of changes in plant biochemical pathways on the basis of changed gene expression profile**. A web based tool, Plant MetGenMap (http://bioinfo.bti.cornell.edu/cgi-bin/MetGenMAP/home.cgi) was used to predict the changes. **(A)** Glutathione mediated detoxification of xenobiotics. **(B)** Flavones and derivative biosynthetic pathway. Red color shows the upregulation of involved genes while green shows down. Please see Materials and Methods for more detail.

To visualize the effect of AuCl^−^_4_ in the context of metabolic pathways, we analyzed the DEGs using web-based analysis program, Plant MetGenMap (http://bioinfo.bti.cornell.edu/cgi-bin/MetGenMAP/home.cgi) and Plant Metabolic Network (http://pmn.plantcyc.org). Glutathione mediated detoxification, flavones and derivative biosynthetic, ethylene biosynthetic, cytokinins-O-glucoside biosynthetic, jasmonic acid biosynthetic, and terpenoids pathways were predicted to be significantly changed (Figure 5, Supplementary Figures 2, 3, Supplementary File 5). It appears that the glutathione conjugation was used as a primary tool to cope up with toxicity. Flavones and derivative biosynthetic pathways were predicted to be upregulated. Up-regulation of glucosyltransferases indicates the glucosylation of compounds, which could change the biological activity significantly. Flavones like quercetin and Kaempferol possess antioxidative properties, thus they might reduce the stress effects (Winkel-Shirley, 2002). We predicted glucosylation of cytokinins leading to the inhibition of cytokinin activity (Supplementary Figure 2), as in an earlier study (Hou et al., 2004). Similarly ABA was predicted to be conjugated with glucose, turning it into an inactive form (Supplementary Figure 2). These modifications are often required to maintain a fine tuned equilibrium between active and inactive forms of the plant hormones. Interestingly, lutein (terpenoids) biosynthesis was also predicted to be upregulated. It is reported to have unique medicinal properties and used as an antioxidant (Tian et al., 2004). Overall, these biochemical predictions indicate a fine-tuned regulation of secondary metabolites to address the challenge posed by the AuCl^−^_4_ treatment.

### Identification of over-represented motifs in upstream sequences of DEGs in response to AuCl^−^_4_

**Figure 6:**
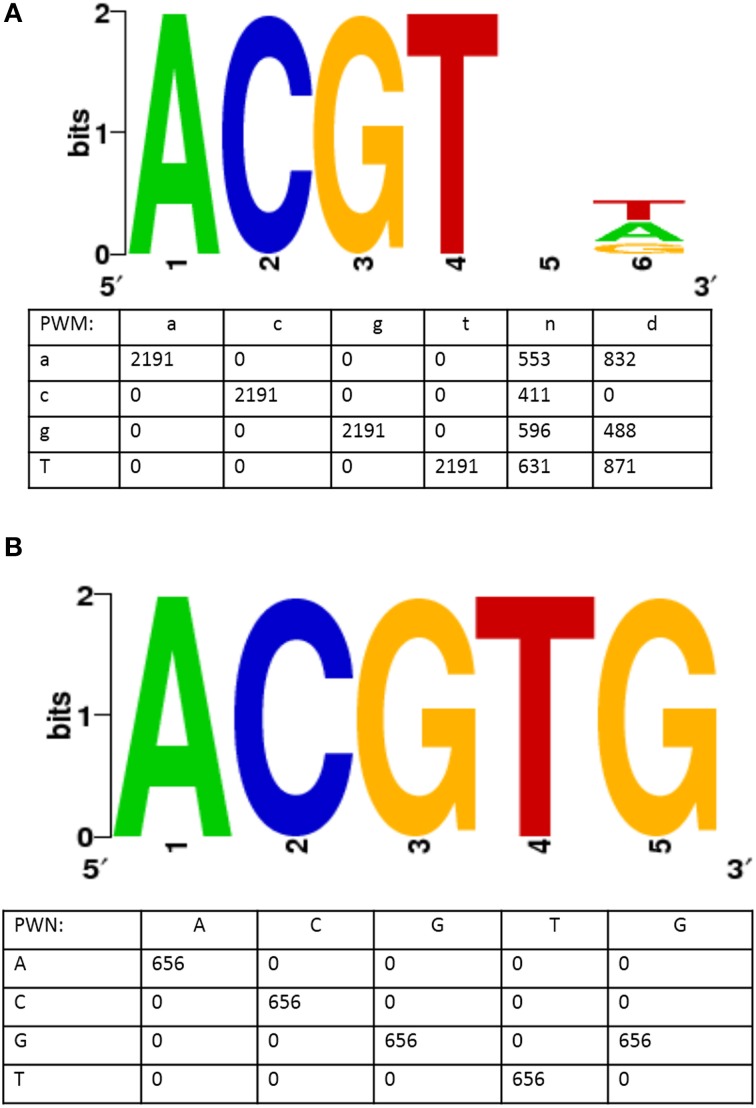
**ABA Responsive cis-regulatory element (ABRE) identified in upstream region of genes upregulated during AuCl^−^_4_ treatment**. Figure shows two statistically highly significant consensus sequence generated by a motif finder tool, SCOPE (http://genie.dartmouth.edu/scope/). **(A)** It shows a motif similar to ABA responsive core element. **(B**) It shows a motif representing a complete ABA responsive element. The numeric values in the position weight metrix (PWMs) indicates the number of times a particular base present in the respective site.

The question of identification of over-represented cis-regulatory elements is important as it sheds light on the regulatory mechanism active in the particular environmental condition. To address this, we pulled out the 1000 bp upstream sequences of the up- or downregulated genes from the Tair10 database and submitted them to the SCOPE motif finder tool (http://genie.dartmouth.edu/scope/) to identify the enriched motifs. We identified an abscisic acid response core element, (ABRE): ACGT, in the 1000 bp upstream sequences of 419 upregulated genes (Table 3, Figure 6). The same motif was also identified by the TAIR motif finder tool (http://www.arabidopsis.org/tools/bulk/motiffinder/index.jsp, data not shown). Interestingly, the top two statistically significant motifs represented ABRE cis-regulatory elements (Figure 6). Table 3 describes the general information about the motif. For example, count indicates the number of occurrence of the motif in the input data. Coverage indicates the fraction of genes containing the motif, and the algorithm used to identify the motif.

**Table 3:** **Summary features of 5 most significant motifs enriched by the SCOPE tool**.

**Consensus sequence**	**Count**	**Sig value**	**Coverage (%)**	**Algorithm**
Acgtnd	2191	200.2	94.3	PRISM
Acgtg	656	73.6	69.3	PRISM
Rktcwahv	840	55.0	88.9	PRISM
Aatat	3378	51.3	98.8	PRISM
Ataaa	3764	48.2	99.8	PRISM

ABA, known as an abiotic stress hormone of plants, mediates several signaling reactions in response to altered environmental conditions. The motif ACGT was earlier identified in the regulatory regions of the genes induced by environmental related factors. Moreover, ABA response is associated with Ca^2+^ ion mediated signaling and this motif was reported to be involved in the Ca^2+^ mediated signaling. Recently, it has been demonstrated that the promoter sequences of Ca^2+^ ATPases harbors ABA related cis-acting elements (ABRE), indicating ABA mediated Ca^2+^ ion response (Huda et al., 2013). Taken together, these findings suggest that the AuCl^−^_4_ response is mediated through ABA signaling, modulating the large set of genes for the adaptation purposes.

We also identified the enriched represented motifs in upstream sequences of downregulated genes, as shown in Supplementary Figure 4. However, these motifs were unknown and not assigned to any important known regulatory element or part of the signal transduction mechanisms.

### A hypothetical model displaying AuCl^−^_4_ signaling

**Figure 7:**
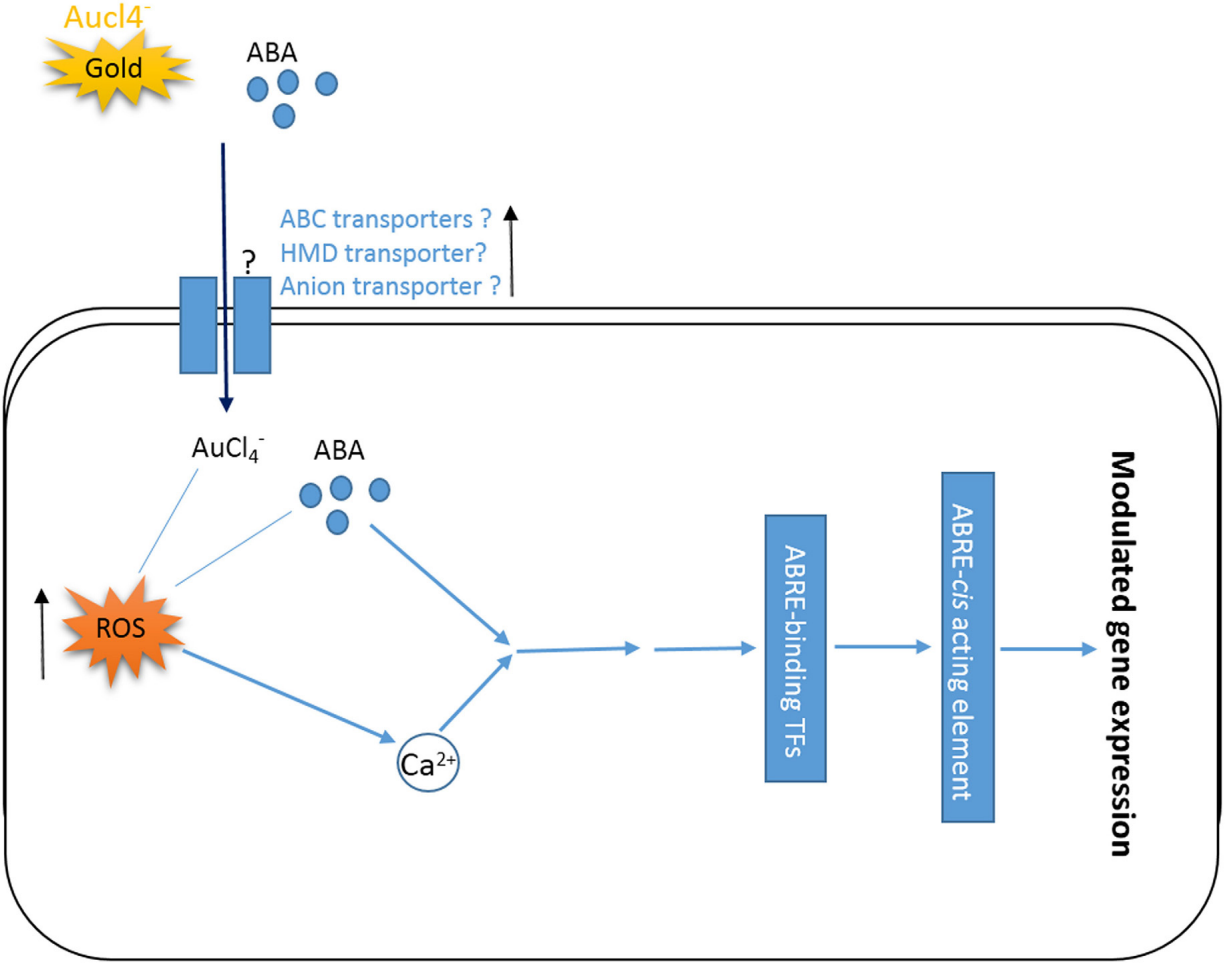
**A hypothetical model displaying ABA mediated signaling during gold (AuCl^−^_4_) exposure**. AuCl^−^_4_ could trigger ABA mediated signaling. AuCl^−^_4_ and or ABA might be taken inside the cells through various transporters and could elevate the level of ROS triggering the changes in Ca^2+^ level. Eventually ABA and/or Ca^2+^ may induce ABRE-binding transcription factors through some intermediates and regulate the global gene expression. ↑Shows the upregulation of related genes.

On the basis of the results obtained in the present work and earlier reported information, a hypothetical model was proposed (Figure 7). This model shows how AuCl^−^_4_ treatment triggers signals modulating the global gene expression in a short period of time. AuCl^−^_4_, like the other heavy metal, may be taken inside the cells through ABC transporters, heavy metal domain containing transporters and/or anion transporters (Chakrabarty et al., 2009; Socha and Guerinot, 2014). ABC transporters are also known for the ABA influx to the plant cells (Fujii, 2014). Both heavy metal (AuCl^−^_4_) and ABA, potentially could increase the level of ROS (Pottosin et al., 2014) and alter the Ca^2+^ level inside the cells, inducing ABRE binding transcription factor (Kaplan et al., 2006; Finkler et al., 2007) and eventually regulating the global gene expression by binding on ABRE element (Galon et al., 2010; Huda et al., 2013).

## Authors' contributions

Devesh Shukla carried out bioinformatics, analyzed the data and drafted the manuscript. Sneha Krishnamurthy performed the experiments and contributed in data discussion. Shivendra V. Sahi contributed intellectually with all the aspect of this research work and helped in finalizing the manuscript.

### Conflict of interest statement

The authors declare that the research was conducted in the absence of any commercial or financial relationships that could be construed as a potential conflict of interest.

## Abbreviations

DEGs: Differentially expressed genes
As^V^: Arsenate
Cd: Cadmium

## References

[B1] Barbez E. Kubeš M. Rolčík J. Béziat C. Pěnčík A. Wang B.. (2012). A novel putative auxin carrier family regulates intracellular auxin homeostasis in plants. Nature 485, 119–122. 10.1038/nature11001 22504182

[B2] Besseau S. Li J. Palva E. T. (2012). WRKY54 and WRKY70 co-operate as negative regulators of leaf senescence in *Arabidopsis thaliana*. J. Exp. Bot. 63, 2667–2679. 10.1093/jxb/err450 22268143 PMC3346227

[B3] Cao M. J. Wang Z. Wirtz M. Hell R. Oliver D. J. Xiang C. B. (2013). SULTR3;1 is a chloroplast-localized sulfate transporter in *Arabidopsis thaliana*. Plant J. 73, 607–616. 10.1111/tpj.12059 23095126

[B4] Chakrabarty D. Trivedi P. K. Misra P. Tiwari M. Shri M. Shukla D.. (2009). Comparative transcriptome analysis of arsenate and arsenite stresses in rice seedlings. Chemosphere 74, 688–702. 10.1016/j.chemosphere.2008.09.082 18996570

[B5] Chen P. C. Mwakwari S. C. Oyelere A. K. (2008). Gold nanoparticles: from nanomedicine to nanosensing. Nanotechnol. Sci. Appl. 1, 45–66. 10.2147/NSA.S3707 24198460 PMC3781743

[B6] Christiansen M. W. Gregersen P. L. (2014). Members of the barley NAC transcription factor gene family show differential co-regulation with senescence-associated genes during senescence of flag leaves. J. Exp. Bot. 65, 4009–4022. 10.1093/jxb/eru046 24567495 PMC4106437

[B7] Finkler A. Ashery-Padan R. Fromm H. (2007). CAMTAs: calmodulin-binding transcription activators from plants to human. FEBS Lett. 581, 3893–3898. 10.1016/j.febslet.2007.07.051 17689537

[B8] Fujii H. (2014). Abscisic acid implication in plant growth and stress responses, in Phytohormones: A Window to Metabolism, Signaling and Biotechnological Applications, eds Tran L. S. P. Pal S. (New York, NY: Springer), 37–54.

[B9] Fujiki Y. Yoshikawa Y. Sato T. Inada N. Ito M. Nishida I.. (2001). Dark-inducible genes from *Arabidopsis thaliana* are associated with leaf senescence and repressed by sugars. Physiol. Plant 111, 345–352. 10.1034/j.1399-3054.2001.1110312.x 11240919

[B10] Galon Y. Finkler A. Fromm H. (2010). Calcium-regulated transcription in plants. Mol. Plant 3, 653–669. 10.1093/mp/ssq019 20457642

[B11] Geetha R. Ashokkumar T. Tamilselvan S. Govindaraju K. Mohamed Sadiq A. Singaravelu G. (2013). Green synthesis of gold nanoparticles and their anticancer activity. Cancer Nanotech. 4, 91–98 10.1007/s12645-013-0040-9 PMC4451866 26069504

[B12] Hou B. Lim E. K. Higgins G. S. Bowles D. J. (2004). N–glucosylation of Cytokinins by glycosyltransferases of *Arabidopsis thaliana*. J. Biol. Chem. 279, 47822–47832. 10.1074/jbc.M409569200 15342621

[B13] Huda K. M. K. Banu M. S. A. Pathi K. M. Tuteja N. (2013). Reproductive organ and vascular specific promoter of the rice plasma membrane Ca^2+^ ATPase mediates environmental stress responses in plants. PLoS ONE 8:e57803. 10.1371/journal.pone.0057803 23469243 PMC3585799

[B14] Jain A. Poling M. D. Smith A. P. Nagarajan V. K. Lahner B. Meagher R. B.. (2009). Variations in the composition of gelling agents affect morphophysiological and molecular responses to deficiencies of phosphate and other nutrients. Plant Physiol. 150, 1033–1049. 10.1104/pp.109.136184 19386810 PMC2689959

[B15] Jain A. Sinilal B. Starnes D. L. Sanagala R. Krishnamurthy S. Sahi S. V. (2014). Role of Fe-responsive genes in bioreduction and transport of ionic gold to roots of *Arabidopsis thaliana* during synthesis of gold nanoparticles. Plant Physiol. Biochem. 84, 189–196. 10.1016/j.plaphy.2014.09.013 25289518

[B16] Johnston C. W. Wyatt M. A. Li X. Ibrahim A. Shuster J. Southam G.. (2013). Gold biomineralization by a metallophore from a gold-associated microbe. Nat. Chem. Biol. 9, 241–243. 10.1038/nchembio.1179 23377039

[B17] Kang J. Hwang J. U. Lee M. Kim Y. Y. Assmann S. M. Martinoia E.. (2010). PDR-type ABC transporter mediates cellular uptake of the phytohormone abscisic acid. Proc. Natl. Acad. Sci. U.S.A. 107, 2355–2360. 10.1073/pnas.0909222107 20133880 PMC2836657

[B18] Kaplan B. Davydov O. Knight H. Galon Y. Knight M. R. Fluhr R.. (2006). Rapid transcriptome changes induced by cytosolic Ca^2+^ transients reveal ABRE-related sequences as Ca^2+^-responsive cis elements in *Arabidopsis*. Plant Cell 18, 2733–2748. 10.1105/tpc.106.042713 16980540 PMC1626612

[B19] Kumar V. Yadav S. K. (2009). Plant-mediated synthesis of silver and gold nanoparticles and their applications. J. Chem. Tech. Biotech. 84, 151–157 10.1002/jctb.2023

[B20] Lee E. J. Matsumura Y. Soga K. Hoson T. Koizumi N. (2007). Glycosyl hydrolases of cell wall are induced by sugar starvation in *Arabidopsis*. Plant Cell Physiol. 48, 405–413. 10.1093/pcp/pcm009 17234672

[B21] Lee M. Lee K. Lee J. Noh E. W. Lee Y. (2005). AtPDR12 contributes to lead resistance in *Arabidopsis*. Plant Physiol. 138, 827–836. 10.1104/pp.104.058107 15923333 PMC1150400

[B22] Li L. He Z. Pandey G. K. Tsuchiya T. Luan S. (2002). Functional cloning and characterization of a plant efflux carrier for multidrug and heavy metal detoxification. J. Biol. Chem. 277, 5360–5368. 10.1074/jbc.M108777200 11739388

[B23] Limbach L. K. Wick P. Manser P. Grass R. N. Bruinink A. Stark W. J. (2007). Exposure of engineered nanoparticles to human lung epithelial cells: influence of chemical composition and catalytic activity on oxidative stress. Environ. Sci. Tech. 41, 4158–4163. 10.1021/es062629t 17612205

[B24] Liu F. Jiang H. Ye S. Chen W. P. Liang W. Xu Y.. (2010). The *Arabidopsis* P450 protein CYP82C2 modulates jasmonate-induced root growth inhibition, defense gene expression and indole glucosinolate biosynthesis. Cell Res. 20, 539–552. 10.1038/cr.2010.36 20354503

[B25] Livak K. J. Schmittgen T. D. (2001). Analysis of relative gene expression data using real-time quantitative PCR and the 2(-Delta Delta C(T) methods. Methods 25, 402–408. 10.1006/meth.2001.1262 11846609

[B26] Mars K. A. (1996). The functions and regulation of glutathione-S-transferases in plants. Annu. Rev. Plant Physiol. Plant Mol. Biol. 47, 127–158. 10.1146/annurev.arplant.47.1.127 15012285

[B27] Mishra M. Kanwar P. Singh A. Pandey A. Kapoor S. Pandey G. K. (2013). Plant omics: genome-wide analysis of ABA Repressor1 (ABR1) related genes in rice during abiotic stress and development. Omics J. Int. Biol. 17, 439–450. 10.1089/omi.2012.0074 23895290

[B28] Mishra S. Shukla A. Upadhyay S. Sharma P. Singh S. Phukan U. J.. (2014). Identification, occurrence, and validation of DRE and ABRE cis-regulatory motifs in the promoter regions of genes of *Arabidopsis thaliana*. J. Int. Plant Biol. 56, 388–399. 10.1111/jipb.12149 24581225

[B29] Niemietz C. M. Tyerman S. D. (2002). New potent inhibitors of aquaporins: silver and gold compounds inhibit aquaporins of plant and human origin. FEBS. Let. 531, 443–447. 10.1016/S0014-5793(02)03581-0 12435590

[B30] Nitta Y. Ding P. Zhang Y. (in press). Heterotrimeric G proteins in plant defense against pathogens and aba signaling. Environ. Exp. Bot. 10.1016/j.envexpbot.2014.06.01119054360

[B31] Pandey G. K. Grant J. J. Cheong Y. H. Kim B. G. Li L. Luan S. (2005). ABR1, an APETALA2-domain transcription factor that functions as a repressor of ABA response in *Arabidopsis*. Plant Physiol. 139, 1185–1193. 10.1104/pp.105.066324 16227468 PMC1283757

[B32] Pottosin I. Velarde-Buendía A. M. Bose J. Zepeda-Jazo I. Shabala S. Dobrovinskaya O. (2014). Cross-talk between reactive oxygen species and polyamines in regulation of ion transport across the plasma membrane: implications for plant adaptive responses. J. Exp. Bot. 65, 1271–1283. 10.1093/jxb/ert423 24465010

[B33] Reith F. Etschmann B. Grosse C. Moors H. Benotmane M. A. Monsieurs P.. (2009). Mechanisms of gold biomineralization in the bacterium Cupriavidus metallidurans. Proc. Natl. Acad. Sci. U.S.A. 106, 17757–17762. 10.1073/pnas.0904583106 19815503 PMC2764933

[B34] Ren X. Chen Z. Liu Y. Zhang H. Zhang M. Liu Q.. (2010). ABO3, a WRKY transcription factor, mediates plant responses to abscisic acid and drought tolerance in *Arabidopsis*. Plant J. 63, 417–429. 10.1111/j.1365-313X.2010.04248.x 20487379 PMC3117930

[B35] Rodriguez E. Parsons J. G. Peralta-Videa J. R. Cruz-Jimenez G. Romero-Gonzalez J. Sanchez-Salcido B. E.. (2007). Potential of Chilopsis linearis for gold phytomining: using XAS to determine gold reduction and nanoparticle formation within plant tissues. Int. J. Phyto. 9, 133–147. 10.1080/15226510701232807 18246721

[B36] Schluttenhofer C. Pattanaik S. Patra B. Yuan L. (2014). Analyses of Catharanthus roseus and *Arabidopsis thaliana* WRKY transcription factors reveal involvement in jasmonate signaling. BMC Genomics 15:502. 10.1186/1471-2164-15-502 24950738 PMC4099484

[B37] Sharma N. C. Sahi S. V. Nath S. Parsons J. G. Gardea-Torresdey J. L. Pal T. (2007). Synthesis of plant-mediated gold nanoparticles and catalytic role of biomatrix-embedded nanomaterials. Environ. Sci. Tech. 41, 5137–5142. 10.1021/es062929a 17711235 PMC2518977

[B38] Shibuya K. Shimizu K. Niki T. Ichimura K. (2014). Identification of NAC transcription factor, EPHEMERAL1, that controls petal senescence in Japanese morning glory. Plant J. Cell Mol. Biol. 79, 1044–1051. 10.1111/tpj.12605 24961791

[B39] Shukla D. Kesari R. Mishra S. Dwivedi S. Tripathi R. D. Nath P.. (2012). Expression of phytochelatin synthase from aquatic macrophyte Ceratophyllum demersum L. enhances cadmium and arsenic accumulation in tobacco. Plant Cell Rep. 31, 1687–1699. 10.1007/s00299-012-1283-3 22614255

[B40] Shukla D. Kesari R. Tiwari M. Dwivedi S. Tripathi R. D. Nath P.. (2013b). Expression of Ceratophyllum demersum phytochelatin synthase, CdPCS1, in *Escherichia coli* and *Arabidopsis* enhances heavy metal(loid)s accumulation. Protoplasma 250, 1263–1272. 10.1007/s00709-013-0508-9 23702817

[B41] Shukla D. Tiwari M. Tripathi R. D. Nath P. Trivedi P. K. (2013a). Synthetic phytochelatins complement a phytochelatin-deficient *Arabidopsis* mutant and enhance the accumulation of heavy metal(loid)s. Biochem. Biophy. Res. Comm. 434, 664–669. 10.1016/j.bbrc.2013.03.138 23587904

[B42] Socha A. L. Guerinot M. L. (2014). Mn-euvering manganese: the role of transporter gene family members in manganese uptake and mobilization in plants. Front. Plant Sci. 5:106. 10.3389/fpls.2014.00106 24744764 PMC3978347

[B43] Spivak M. Y. Bubnov R. V. Yemets I. M. Lazarenko L. M. Tymoshok N. O. Ulberg Z. R. (2013). Development and testing of gold nanoparticles for drug delivery and treatment of heart failure: a theranostic potential for PPP cardiology. EPMA. J. 4:20. 10.1186/1878-5085-4-20 23889805 PMC3751918

[B44] Starnes D. L. Jain A. Sahi S. V. (2010). In planta engineering of gold nanoparticles of desirable geometries by modulating growth conditions: an environment-friendly approach. Environ. Sci. Tech. 44, 7110–7115. 10.1021/es101136q 20698550

[B45] Takase T. Nakazawa M. Ishikawa A. Manabe K. Matsui M. (2003). DLF 2, a new member of the *Arabidopsis* GH3 gene family, is involved in red light-specific hypocotyl elongation. Plant Cell Physiol. 44, 1071–1080. 10.1093/pcp/pcg130 14581632

[B46] Taylor A. F. Rylott E. L. Anderson C. W. Bruce N. C. (2014). Investigating the toxicity, uptake, Nanoparticle formation and genetic response of plants to gold. PLoS ONE 9:e93793. 10.1371/journal.pone.0093793 24736522 PMC3988041

[B47] Tian L. Musetti V. Kim J. Magallanes-Lundback M. DellaPenna D. (2004). the *Arabidopsis* LUT1 locus encodes a member of the cytochrome p450 family that is required for carotenoid epsilon-ring hydroxylation activity. Proc. Natl. Acad. Sci. U.S.A. 101, 402–407. 10.1073/pnas.2237237100 14709673 PMC314197

[B48] Tiwari M. Sharma D. Singh M. Tripathi R. D. Trivedi P. K. (2014). Expression of OsMATE1 and OsMATE2 alters development, stress responses and pathogen susceptibility in *Arabidopsis.* Sci. Rep. 4:3964. 10.1038/srep03964 24492654 PMC3912489

[B49] Tripathi P. Rabara R. C. Rushton P. J. (2014). A systems biology perspective on the role of WRKY transcription factors in drought responses in plants. Planta 239, 255–266. 10.1007/s00425-013-1985-y 24146023

[B50] Wagner U. Edwards R. Dixon D. P. Mauch F. (2002). Probing the diversity of the *Arabidopsis* glutathione S-transferase gene family. Plant Mol. Biol. 49, 515–532. 10.1023/A:1015557300450 12090627

[B51] Winkel-Shirley B. (2002). Biosynthesis of flavonoids and effects of stress. Curr. Opin. Plant Biol. 5, 218–223. 10.1016/S1369-5266(02)00256-X 11960739

[B52] Yadav D. K. Shukla D. Tuteja N. (2013). Rice heterotrimeric G-protein alpha subunit (RGA1): *in silico* analysis of the gene and promoter and its upregulation under abiotic stress. Plant Physiol. Biochem. 63, 262–271. 10.1016/j.plaphy.2012.11.031 23313793

[B53] Yadav D. K. Shukla D. Tuteja N. (2014). Isolation, *in silico* characterization, localization and expression analysis of abiotic stress-responsive rice G-protein beta subunit (RGB1). Plant Sig. Behav. 9:e28890. 10.4161/psb.28890 24739238 PMC4091194

[B54] Yeh Y. C. Creran B. Rotello V. M. (2012). Gold nanoparticles: preparation, properties, and applications in bionanotechnology. Nanoscale 4, 1871–1880. 10.1039/c1nr11188d 22076024 PMC4101904

