# Supplementary material for: Genome wide transcriptome analysis reveals ABA mediated response in Arabidopsis during gold (AuCl−4) treatment
Source: Front Plant Sci. 2014 Nov 28;5:652. doi: 10.3389/fpls.2014.00652 (PMC4246665; doi:10.3389/fpls.2014.00652)
Supplement: Supplementary file 1 [file Presentation1.ZIP › Supplementary material/File List.DOCX]

***Supplementary Material***

**Genome Wide Transcriptome Analysis reveals ABA mediated response in Arabidopsis during Gold (AuCl_4_^-^) treatment**

**Devesh Shukla, Sneha Krishnamurthy, Shivendra V. Sahi***

Department of Biology, Western Kentucky University, 1906 College heights, Bowling Green, 42101-1080 Kentucky, U.S.A.

*Correspondence: Dr Shivendra Sahi, Department of Biology, Western Kentucky University Bowling Green, KY, 42101-1080, USA

[shiv.sahi@wku.edu](mailto:shiv.sahi@wku.edu)

1. **Supplementary Data**
   1. Supplementary File 1: List of DEGs in response to AuCl_4_^-^
   2. Supplementary File 2: List of DEEs in response to AuCl_4_^-^
   3. Supplementary File 3: List of significant enriched GO term
   4. Supplementary File 4: List of gene selected for heat map
   5. Supplementary File 5: Plant MetGenMap input File
   6. Supplementary File 6: WebScope xml file for upstream regions of upregulated genes
   7. Supplementary File 7: WebScope xml file for upstream regions of downregulated genes
2. **Supplementary Figures and Tables**
   1. **Supplementary Table 1** : List of primers used for qRT-PCR
   2. **Supplementary Figures**

**Supplementary Figure 1a.** **Biological process hieratical graph display for the enriched GO terms belonging to differentially regulated genes using agriGO.** The colour of the boxes indicates the level of significance. The darker (red) is more significant, while white is not. The information inside the box includes GO term, p-value, GO description, item number, mapping the GO in the query list and background and total number of query list and background. The p value represents the statistical significance of the term. If the q-value was higher than the cutoff (p<0.05), only GO information is given in the boxes.The colour of the arrows shows the relationship among the GO terms.

**Supplementary Figure 1b**. **Molecular function hieratical graph display for the enriched GO terms of differentially expressed genes using agriGO.** Molecular function. The other details are the same as described in the caption for Figure S1 a.

**Supplementary Figure 2. Prediction of changes in plant biochemical pathways in the context of gene expression profile of AuCl_4_^-^ using the web based tool, Plant MetGenMap (http://bioinfo.bti.cornell.edu/cgi-bin/MetGenMAP/home.cgi).** A, Cytokinins-O-glucoside biosynthesis; B, Ethylene biosynthesis; C, Auxin biosynthesis; D, Abscisic acid modification or inactivation. Red color shows the upregulation of involved genes while green shows down. Please see material and methods for detail.

**Supplementary Figure 3. Prediction for changes in plant biochemical pathways in the context of gene expression profile of AuCl_4_^-^ using the web based tool, Plant MetGenMap (**http://bioinfo.bti.cornell.edu/cgi-bin/MetGenMAP/home.cgi**).** A, Jasmonic acid biosynthesis; B, Lutein biosynthesis; C, Marneral biosynthesis. Red color shows the upregulation of involved genes while green shows down. Please see material and methods for detail.

**Supplementary Figure 4. Over-represented motif identified in upstream sequences of downregulated genes during AuCl_4_^-^ treatment.** Figure shows two statistically highly significant consensus sequence generated by a motif finder tool, SCOPE (http://genie.dartmouth.edu/scope/). A, shows an unknown motif, wnhanta; B, shows a motif, AATTA. The numeric values in the position weight metrix (PWMs) indicates the number of times a particular base present in the respective site.
