# Supplementary material for: Genome wide transcriptome analysis reveals ABA mediated response in Arabidopsis during gold (AuCl−4) treatment
Source: Front Plant Sci. 2014 Nov 28;5:652. doi: 10.3389/fpls.2014.00652 (PMC4246665; doi:10.3389/fpls.2014.00652)
Supplement: Supplementary file 1 [file Presentation1.ZIP › Supplementary material/Supplementary Table 1.DOCX]

Supplementary Table 1: List of primers used in this study

| Primer | Sequence (5' to 3') | Purpose |
| --- | --- | --- |
| AT3G60140.1F | CCAATCGAGGAGAGATGAGGAGA | Q-RT PCR |
| AT3G60140.1R | AGATGGGTTCTCTATGCCTTGC |  |
| AT4G31970.1 F | GGATATGCCTGTTGATATGACTGAG |  |
| AT4G31970.1 R | CACATAAAGCCCTTCCTTAAGACG |  |
| AT3G13630.2F | GGATATGAGATTGGAAAAGGAAGCC |  |
| AT3G13630.2R | GGTGATCATAGGGAGAGAGAGTC |  |
| AT1G17170.1 F | GCCTGAGTCAGAGAAGGTCATTAC |  |
| AT1G17170.1 R | CAAAGTAGAGATTTACTCCAACCCAAG |  |
| AT1G15520.1F | GGCAGATAGTAATATGAGTGTGAAGC |  |
| AT1G15520.1R | CAAAGGAAAGATGACGTTCATGGCG |  |
| AT1G71140.1F | GCTCGGTCTTATCGTAATCCTCAC |  |
| AT1G71140.1R | CTCTTCATACTCATCACCCATCACTC |  |
| AT5G37970.1F | CACTTTGGAGAAGGTGTGGTCAAC |  |
| AT5G37970.1R | GGGTACTTGTCGAGCCTTTTAGC |  |
| AT1G17180.1F | GAGTGTGGCTAAGTCTCTTCCTGAT |  |
| AT1G17180.1R | CAGAGCAGAAACAGCAACACAAACAG |  |
| AT2G02930.1F | GCGTCTGCCTCATTGCCCAGTTTCT |  |
| AT2G02930.1R | CTCTCAAGAACGTACACACACTCAC |  |
| AT1G71520.1 F | CTCCGTGTATGATTATCTTGAAGACG |  |
| AT1G71520.1R | GTAAACAGATCCTAACCCTTTGCAC |  |
| AT5G13370.1 F | GAGGGCAGGGGAGGAGAACAAT |  |
| AT5G13370.1 R | GAAGAACAGAGCATTTGTTGGCTGG |  |
| AT1G76520.1F | CTCACTGTTTGGCCAACATTCTTC |  |
| AT1G76520.1 R | CCAATCATCTGGAAGTCCAAGAAC |  |
| AT1G01480.1F | GACGACTTTACGAGGATGGTC |  |
| AT1G01480.1R | TCCAATCTCTCAGACGTTATGCC |  |
| AT1G18830.1 F | CACTCAGCTCTGTCAAGCTCTGGA |  |
| AT1G18830.1R | CGCACATTTTGCCTTCCCGTGACG |  |
| AtTubulin beta 1 F | CTCACAGTCCCGGAGCTGACAC |  |
| At Tubulin beta 1R | GCTTCAGTGAACTCCATCTCGT |  |
