# Supplementary figures and images for: Genome wide transcriptome analysis reveals ABA mediated response in Arabidopsis during gold (AuCl−4) treatment
Source: Front Plant Sci. 2014 Nov 28;5:652. doi: 10.3389/fpls.2014.00652 (PMC4246665; doi:10.3389/fpls.2014.00652)

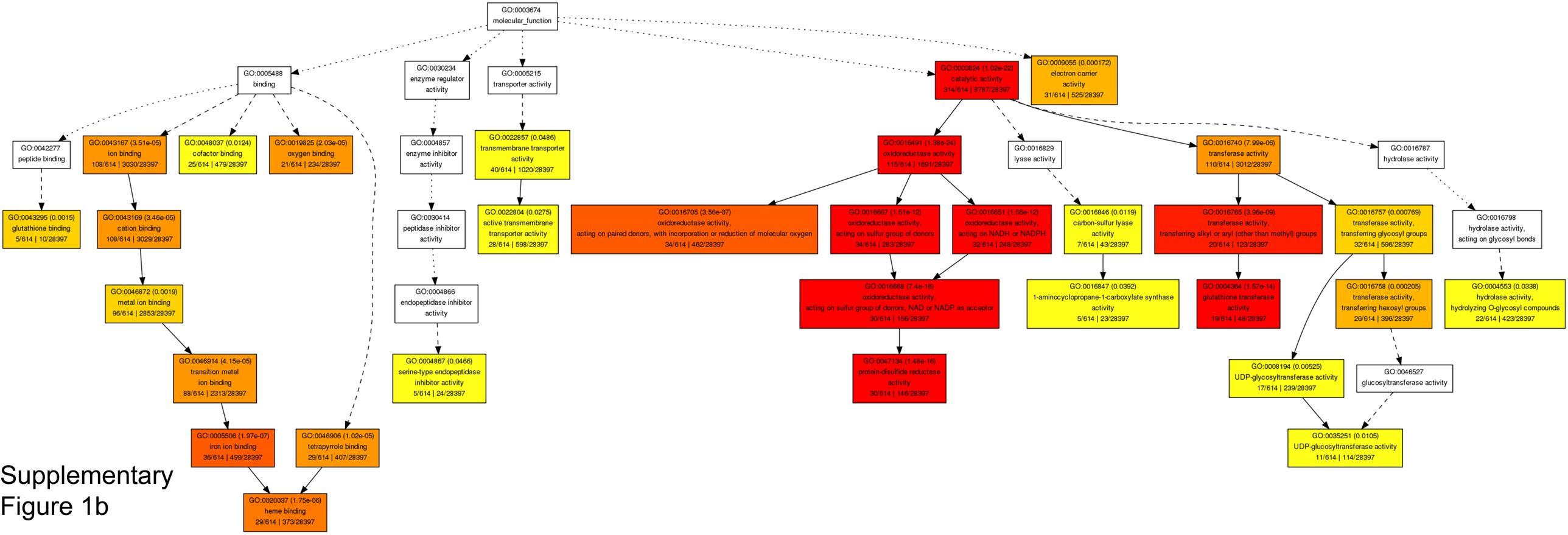

Supplement: Supplementary file 1 [file Presentation1.ZIP › Supplementary material/Supplementary Figure 1b.TIF]

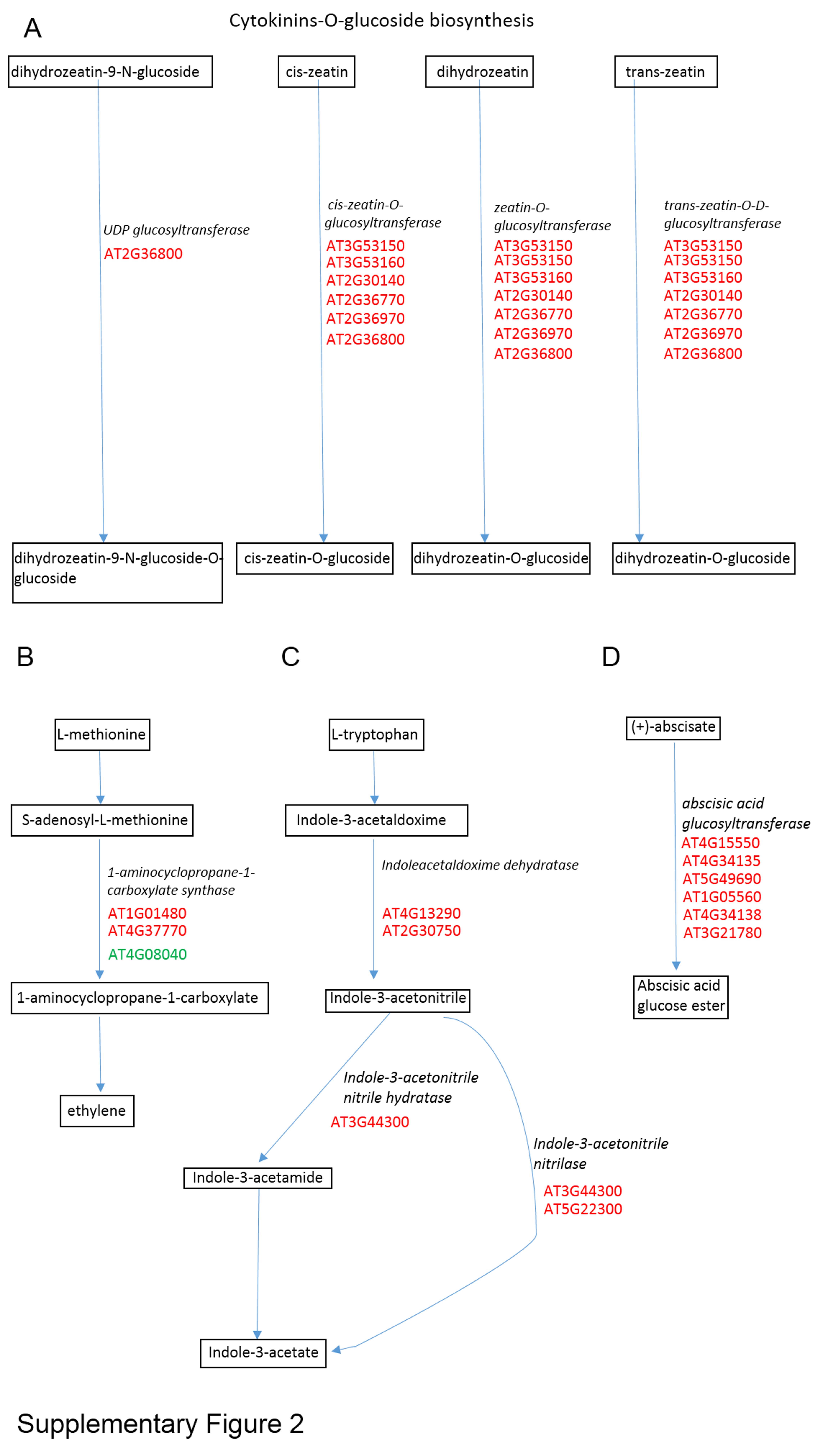

Supplement: Supplementary file 1 [file Presentation1.ZIP › Supplementary material/Supplementary Figure 2.TIF]

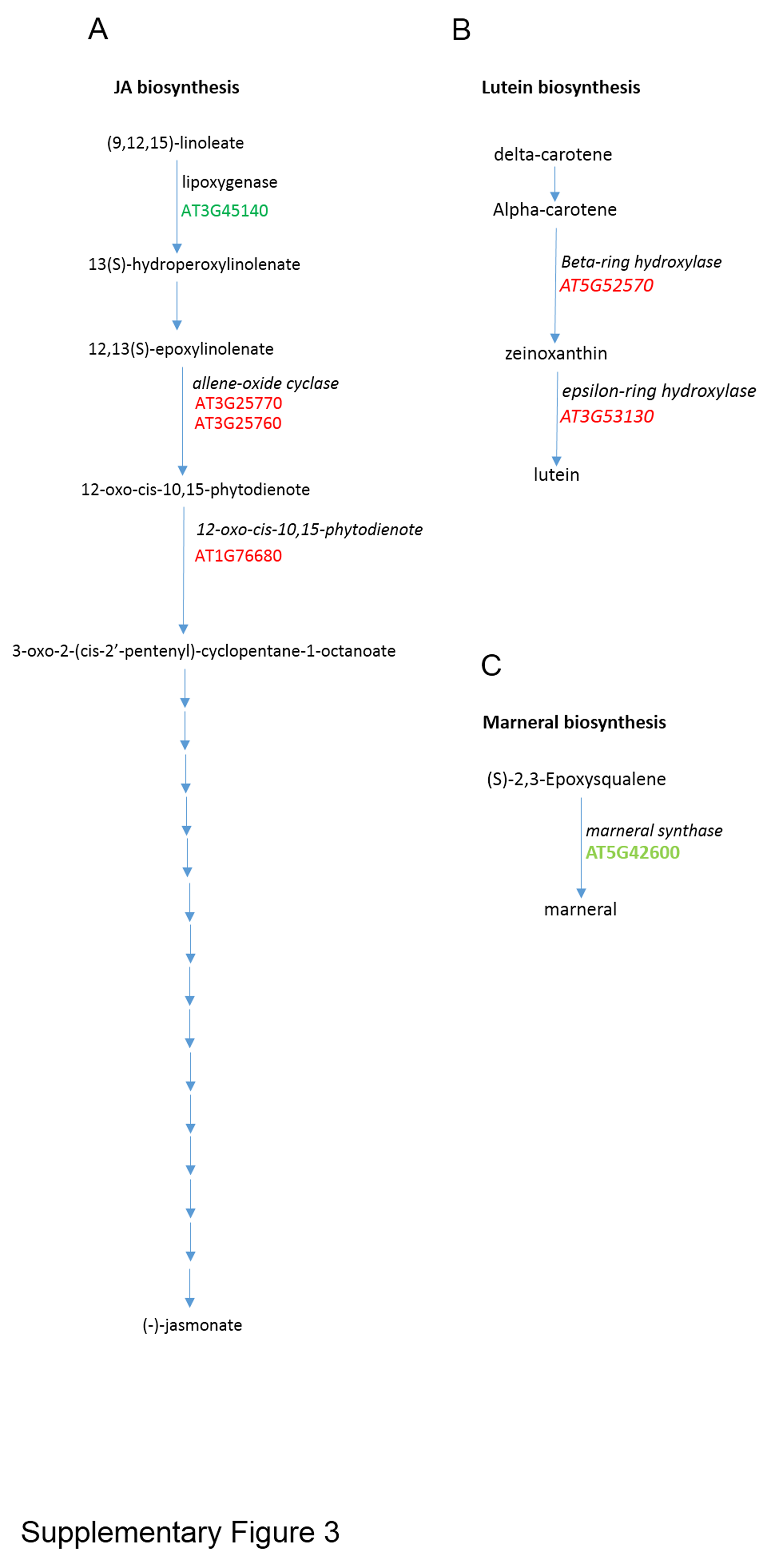

Supplement: Supplementary file 1 [file Presentation1.ZIP › Supplementary material/Supplementary Figure 3.TIF]

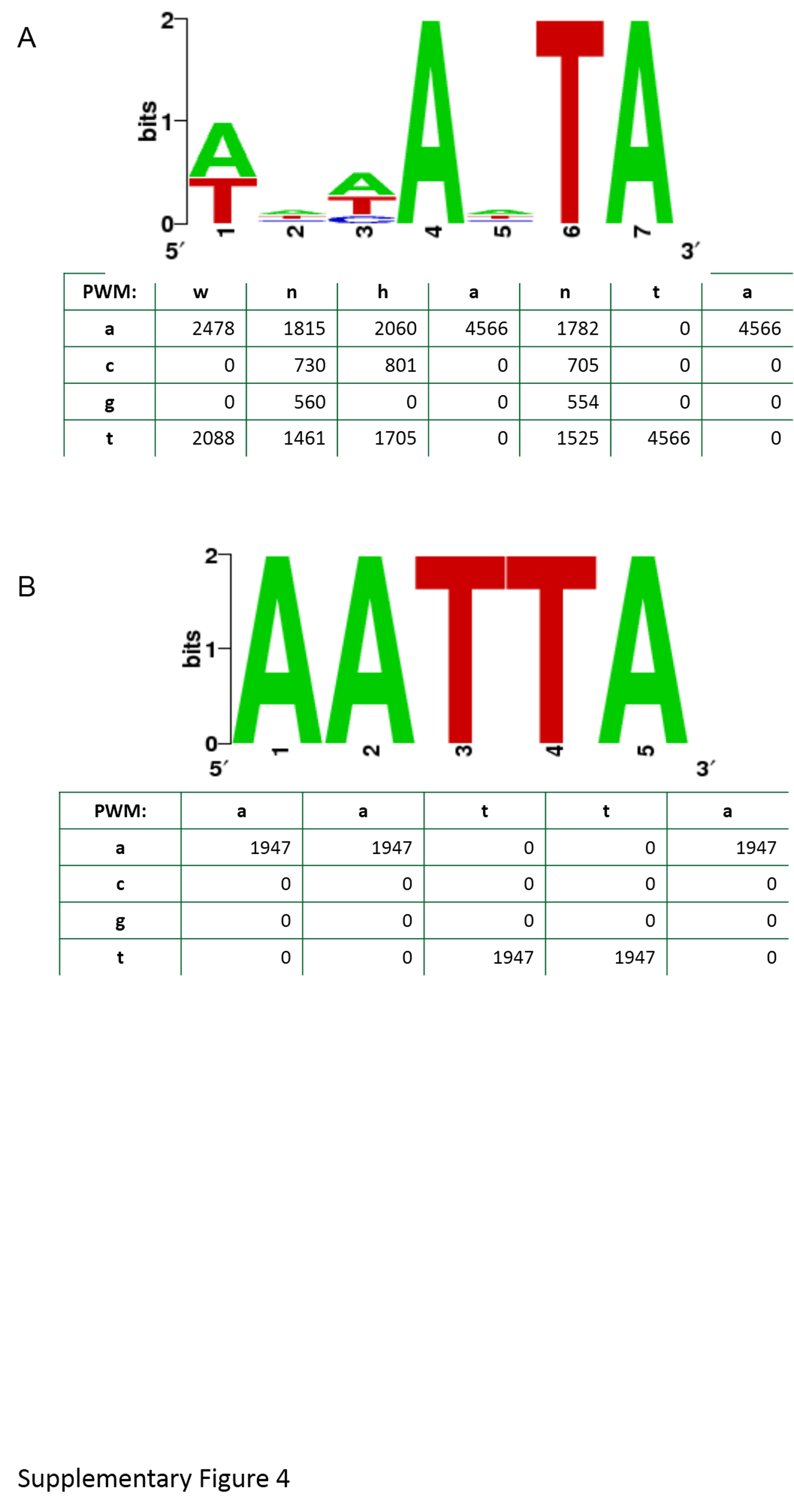

Supplement: Supplementary file 1 [file Presentation1.ZIP › Supplementary material/Supplementary Figure 4.TIF]
